# Stimulant induced Dermatological and Vascular Complications in patients with ADHD: A literature review

**DOI:** 10.1192/j.eurpsy.2022.1066

**Published:** 2022-09-01

**Authors:** F. Arain, M. Jawad, A. Azeem, H. Arain, A. Williams, M. Zeshan

**Affiliations:** 1 BronxCare Health System Icahn School of Medicine at Mount Sinai, Child & Adolescent Psychiatry, Bronx, United States of America; 2 King Edward Medical University, Department Of Psychiatry, Lahore, Pakistan; 3 American School of Doha, Psychiatry, Doha, Qatar; 4 Columbia University, Medicine, NY, United States of America; 5 Touro University California, Psychiatry, California, United States of America; 6 Rutgers New Jersey Medical School, Newark, Psychiatry, NJ, United States of America

**Keywords:** stimulants, vascular complications, ADHD, Reyanaud’s phenomenon

## Abstract

**Introduction:**

Methylphenidate and amphetamine are the two most widely used stimulants in managing Attention Deficit Hyperactivity Disorder (ADHD)^1^. Reynaud’s phenomenon (RP) is a reversible distal vasoconstriction presenting with various dermatological symptoms. RP can secondarily develop after certain medications as well^2^.

**Objectives:**

The review was undertaken to synthesize the incidence of RP within ADHD population treated with stimulants, and any causal relation of RP and stimulant-use.

**Methods:**

PubMed, Psych-Info and Google Scholar were searched using these keywords: skin change, Raynaud, stimulants and methylphenidate. All relevant study types were included. Results were synthesized narratively.

**Results:**

Initial search yielded 240 articles with 5 articles fulfilling our inclusion criteria. One was retrospective case-controlled study while remaining 4 were case reports. Six cases were identified with an age of 12-19 years, who presented with RP after being treated with methylphenidate-or-dextroamphetamine. In one case, multiple clinical signs of RP were seen with cold distal fingers, transient color changes and even frank ulceration^3–6^. In two cases, it was seen that RP was dose-dependent with stimulant use and got resolved after decreasing the dosage respectively. In case-control study, 32 cases with RP and 32 controls were enrolled. The results showed a statistically significant association (χ^2^ =5, p=0.01) between RP and past-or-current stimulant usage.^7^

**Conclusions:**

The literature review suggests weak evidence of the association between RP and stimulant use but no evidence of any causal link. Further studies are needed to identify characters that can predict this adverse effect in vulnerable ADHD individuals.

**Disclosure:**

No significant relationships.

